# Motion Strategy Generation Based on Multimodal Motion Primitives and Reinforcement Learning Imitation for Quadruped Robots

**DOI:** 10.3390/biomimetics11020115

**Published:** 2026-02-04

**Authors:** Qin Zhang, Guanglei Li, Benhang Liu, Chenxi Li, Chuanle Zhu, Hui Chai

**Affiliations:** 1 School of Electrical Engineering, University of Jinan, Jinan 250022, China; 2 School of Control Science and Engineering, Shandong University, Jinan 250061, China

**Keywords:** imitation learning, behavior generation, action design, quadruped robot

## Abstract

With the advancement of task-oriented reinforcement learning (RL), the capability of quadruped robots for motion generation and complex task completion has significantly improved. However, current control strategies require extensive domain expertise and time-consuming design processes to acquire operational skills and achieve multi-task motion control, often failing to effectively manage complex behaviors composed of multiple coordinated actions. To address these limitations, this paper proposes a motion policy generation method for quadruped robots based on multimodal motion primitives and imitation learning. A multimodal motion library was constructed using 3D engine motion design, motion capture data retargeting, and trajectory planning. A temporal domain-based behavior planner was designed to combine these primitives and generate complex behaviors. We developed a RL-based imitation learning training framework to achieve precise trajectory tracking and rapid policy deployment, ensuring the effective application of actions/behaviors on the quadruped platform. Simulation and physical experiments conducted on the Lite3 quadruped robot validated the efficacy of the proposed approach, offering a new paradigm for the deployment and development of motion strategies for quadruped robots.

## 1. Introduction

Quadruped robots exhibit exceptional terrain adaptability, demonstrating broad application prospects in disaster rescue, environmental inspection, and related fields [1,2,3]. A representative example is Boston Dynamics’ Spot, which has shown outstanding performance across diverse environments, including construction sites and disaster zones [4]. However, existing research predominantly focuses on unimodal policy transfer, failing to effectively integrate the complementary advantages of different motion generation methods [5]. Consequently, establishing a cross-modal collaborative framework to achieve motion strategy fusion and adaptive selection is pivotal for advancing quadruped locomotion intelligence.

To enable effective motion strategy fusion, a systematic evaluation of existing motion generation approaches and their limitations is essential. Current research focuses on two primary methodologies: trajectory planning-based and motion capture-based methods.

Trajectory planning-based motion generation aims to produce optimal motion trajectories that satisfy smoothness, feasibility, and energy efficiency under multiple constraints. Medeiros et al. [6] employed nonlinear programming to co-optimize base/wheel positions, interaction forces, and terrain information for wheeled-legged robots. Liu et al. [7] proposed a hierarchical framework combining front-end safety search, B-spline convex hull optimization, and iterative refinement, achieving 100% navigation success in static cluttered environments while reducing energy consumption. Song et al. [8] focused on energy-optimal jumping trajectory planning, enabling robots to overcome complex obstacles.

Motion data-driven generation methods leverage biological motion characteristics to create robust, lifelike, and generalizable quadruped motions. Ju et al. [9] pioneered the cross-validation of spiral theory stability models with biological gait data, systematically revealing the dynamic advantages of common gait sequences. Yao et al. [10] developed a video-based biomimetic adaptation network, using deep learning to extract spatiotemporal key features from animal motions and transferring them via a motion adapter. Motion video tracking captures actions from videos and extracts corresponding trajectories for motion generation. Additionally, motion capture technology provides high-precision motion data. Li et al. [3] adopted multimodal motion primitive encoding to decouple cross-scale motion features from canine multi-terrain motion capture data. Fawcett et al. [11] developed a data-driven template-based hierarchical control method for the real-time planning and control of dynamic quadruped robots.

In recent years, reinforcement learning (RL)-based motion control has emerged as a unified framework for robotic locomotion [12]. RL has become a promising paradigm for developing robust legged movement control strategies [13,14,15], enabling agents to learn motion generation policies directly through environmental interactions [16]. Remarkable achievements include agile behaviors such as balancing, running, jumping, and robust walking under environmental uncertainties [17]. Hwangbo et al. [18] established a sim-to-real transfer framework with data-driven actuator modeling. Bellegarda et al. [19] addressed unstructured terrain disturbances via a hybrid RL framework for dynamic jumping control. Azimi and Hoseinnezhad [20] proposed a hierarchical RL framework to enhance the stability and adaptability of quadruped robots in dynamic environments.

Recent studies explore integrating imitation learning into RL to reduce reward design and unnatural behaviors. Peng et al. [21] pioneered a primitive-fused deep RL paradigm, constructing a bio-inspired transfer framework for cross-domain animal-to-robot motion style conversion. To address skill generalization challenges, Yang et al. [22] proposed a biomimetic motion primitive learning framework with heterogeneous reward mechanisms, enabling robots to acquire diverse skills through imitation learning. Roh [23] designed a ground reaction force (GRF)-based reward function for animal motion imitation, achieving dynamic speed transitions during galloping and validating the efficacy of bio-inspired strategies for dynamic performance optimization. Chen et al. [24] introduced an end-to-end torque control RL paradigm, directly outputting joint torques instead of traditional position control, demonstrating superior anti-disturbance capabilities and reward maximization. Wang et al. [25] abandoned static control for load-carrying quadruped manipulators, proposing an RL-based arm–body dynamic coordination method inspired by quadruped limb synergies, significantly improving disturbance rejection. Miki et al. [26] fused vision and proprioception via gated attention mechanisms, reducing terrain misclassification during Alpine field tests while achieving 0.8 m/s locomotion speeds—their dynamic weighting mechanism offers a novel paradigm for cross-modal collaborative control. Similarly, Ding et al. [27] proposed a vision–language–action model, enabling quadruped robots to perform complex tasks in diverse environments with enhanced adaptability. From the perspective of system modeling assumptions and prior information, existing motion generation and control methods exhibit different trade-offs among interpretability, flexibility, and engineering practicality. Trajectory planning approaches rely on explicit dynamic models and constraints, offering strong interpretability but limited flexibility in complex multi-task scenarios [28]. Reinforcement learning methods optimize policies through reward-driven learning and demonstrate strong adaptability; however, they typically require carefully designed reward functions and extensive interaction data, and their training stability and generalization performance remain challenging in real-world applications [29]. In contrast, imitation learning introduces expert demonstrations as prior knowledge, providing an effective inductive bias for policy search and constraining the optimization process within a reasonable motion manifold [30]. As a result, imitation learning improves training efficiency while maintaining stability and engineering feasibility for complex behavior generation.

Current research on optimizing the locomotion capabilities of quadruped robots often faces challenges, such as limited dimensionality in motion generation, abrupt transitions during behavior composition, and constrained control optimization objectives. These limitations hinder the reliable and efficient execution of smooth movements and multi-task operations in complex scenarios. To address these issues, this paper proposes a motion strategy generation method for quadruped robots based on multimodal motion primitives and imitation learning. The multimodal motion primitives do not refer to multiple motion primitives learned within a unified parameter space. Instead, they denote a collection of heterogeneous action representations derived from distinct motion generation paradigms, including 3D-engine-based keyframe specification, motion primitives obtained via motion capture data retargeting, and analytically generated trajectories based on central pattern generators (CPGs).

Existing approaches are typically trained for a single motion pattern, which makes it difficult to achieve the integrated execution of heterogeneous behaviors—such as stepping in place, locomotion, and posture adjustment—within a unified control framework [18]. In contrast, the proposed method enables a unified representation and seamless switching among multiple behaviors through multimodal motion modeling and a behavior planning mechanism. The main contributions of this paper are as follows:

• A fundamental motion primitive library for quadruped robots was designed, establishing an underlying behavioral foundation for executing complex tasks and enabling flexible motion control.
• A modular architecture was employed to achieve spatiotemporal encoding of motion primitives and skill-chain recombination for quadruped robots, enabling the dynamic synthesis of behavior sequences in complex scenarios through a behavioral planner.
• An expert trajectory-guided Actor–Critic multi-objective optimization framework was improved for the motion control of quadruped robots. It incorporates a composite reward function to achieve hierarchical control under multi-task objectives, while integrating motion primitive imitation learning to accelerate policy convergence during training.

## 2. Methodology

**Figure 1:**
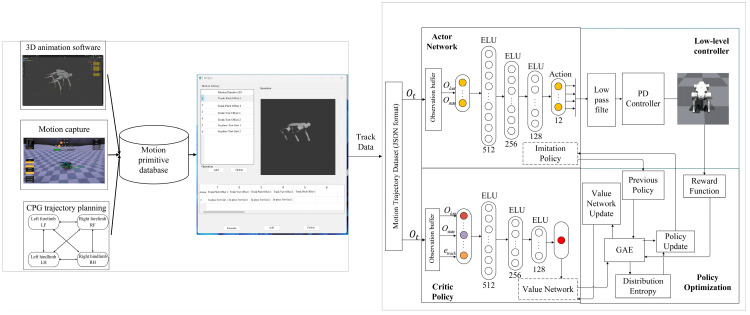
Motion generation and control framework.

Under the robot dynamics constraints and joint-level physical limits, the proposed framework aims to learn a mapping from the robot state space to continuous joint-level control commands, conditioned on the current system state (e.g., joint positions and velocities) and the given motion reference information. The learned policy outputs continuous and physically executable joint-level control signals, which are directly applied to the robot to stably track and compose multiple target motions under physical constraints. As illustrated in Figure 1, the imitation learning-based motion generation and control framework consists of two main components: (1) a motion generation module that produces reference motion sequences for tracking and (2) a motion control module that trains a policy network to achieve high-fidelity reproduction of the target motions.

### 2.1. Behavior Generation

#### 2.1.1. Motion Design Based on a 3D Engine

Utilizing 3D simulation engines for quadruped robot motion design significantly reduces development costs and iteration cycles. Developers can detect and correct motion sequence flaws within the virtual environment. This process primarily consists of three components: modeling and skeletal rigging, keyframe motion design, and motion trajectory extraction and encapsulation, as previously shown in [31].

In the modeling and rigging phase, the kinematically consistent tree is first constructed through topological structuring, followed by geometric validation to ensure global coordinate system alignment and motion consistency. Finally, parametric conversion is performed using a customized plugin to reversely parse the URDF kinematic chain, achieving precise mapping between the skeleton and mechanical topology.

Keyframe motion design deconstructs actions into three phases: initiation, task execution, and termination. Multi-segment Bézier curves are employed to enable smooth transitions and trajectory generation. A custom plugin performs the extraction and encapsulation of motion trajectories. It precisely parses motion data and outputs it in structured CSV-formatted files containing key kinematic parameters such as torso, foot-end, and joint trajectories, along with timestamps.

#### 2.1.2. Motion Capture Data Retargeting Based on Kinematic Chains

**Figure 2:**
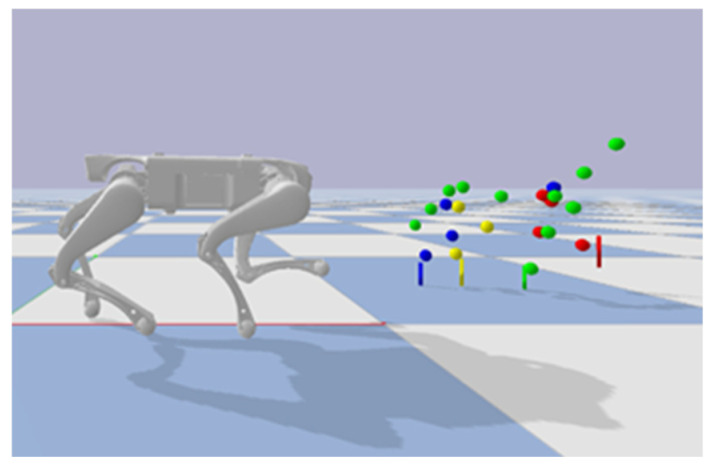
Effectiveness of the size scaling coefficients.

**Figure 3:**
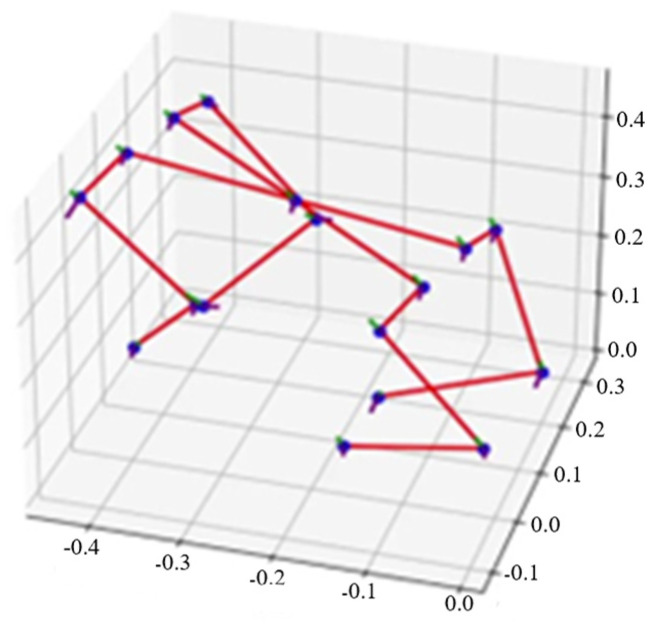
Visualization of the quadruped robot motion data.

To enhance the biomimetic locomotion capabilities of quadruped robots by transferring high-fidelity motion capture data from quadruped animals, we adopt a cross-domain mapping framework based on sampled key points and inverse kinematics (IK), addressing the heterogeneity gap between the motion capture model and the robot. The specific steps are as follows:

• Size scaling: motion capture data was retargeted to the quadruped robot’s kinematic chain through size scaling, eliminating geometric discrepancies between the source character and the target robot. This process calculates segment-specific length ratios to derive independent scaling factors for each kinematic sub-chain. For the quadruped robot utilized in this study, the selected scaling ratio was 0.725, with visual validation illustrated in Figure 2.
• Torso state determination: to resolve misalignment arising from mapping a flexible biological torso to a rigid robotic structure, four naturally symmetric limb connection points were extracted from motion capture data—left/right shoulder joints (anterior) and left/right hip joints (posterior). A rigid-body transformation matrix was computed using the least squares method based on these reference points.
• Key points extraction: biomechanically representative key points were selected for kinematic chain mapping. Shoulder joints (forelimbs) and hip joints (hindlimbs) were extracted as limb root anchor points, corresponding spatially to the base mounting points of the robot’s leg actuators. Their spatial coordinates were directly associated with the relative positional relationships in the torso rigid-body coordinate system, capturing 3D trajectory data of the limb endpoints.
• Inverse kinematics retargeting: biological key-point trajectories were converted into continuous joint-space motion commands. At each timestep t, the source motion specified the 3D position ${\hat{x}}_{i}\left(t\right)$ of key point i. The corresponding target position $x_{i}(q_{t})$ was determined by the robot’s generalized coordinate pose $q_{t}$. Inverse kinematics was then applied to construct a pose sequence $q_{0\;:\;T}$ that tracks key points per frame, satisfying the following:
  (1) $$\arg\min_{q_{0:T}}\sum_{t}\sum_{i}\|{\hat{x}}_{i}(t)-x_{i}(q_{t}){\|}^{2}+{\left(\bar{q}-q_{t}\right)}^{T}W(\bar{q}-q_{t})$$
  where W denotes a diagonal matrix specifying the regularization coefficients for each joint. One set of trajectories is shown in Figure 3.
• Post-processing: joint angle sequences output by inverse kinematics underwent validity screening and smoothing refinement. Simultaneously, centroid position adjustment was performed on motion capture results to ensure the center of mass coincided with the geometric center of the torso’s rigid body.

#### 2.1.3. Trajectory Planning-Based Rhythmic Locomotion Generation for Quadruped Robots

Central Pattern Generators (CPGs) are employed to construct a distributed neural oscillatory network, generating self-stabilizing rhythmic signals through the nonlinear phase dynamics of Hopf oscillator units.

The Hopf oscillator model is defined as follows:

(2) $$\left\{\begin{array}{l} \dot{x}=\alpha (\mu -r^{2})x-\omega y\\ \dot{y}=\alpha (\mu -r^{2})y+\omega x \end{array}\right.$$

where x and y represent the state variables of the Hopf oscillator, whose dimensionality corresponds to the number of leg degrees of freedom. α denotes the convergence rate, μ specifies the oscillation amplitude, r is the intermediate parameter defined as $r^{2}=x^{2}+y^{2}$, and ω determines the steady-state oscillation frequency.

#### 2.1.4. Design of the Behavior Planner

The behavior planner constructs a behavioral decision-making framework, enabling complex behavior generation through modular decomposition and dynamic composition mechanisms. Its core components mainly include:

- An action list management module based on hierarchical dynamic architecture

This module is responsible for converting motion primitives of different modalities and origins into digital objects that can be uniformly parsed and invoked by the system. It employs a hierarchical data structure to decouple and reconstruct key elements of motion primitives, including semantic descriptions, parameter configurations, and execution logic. This process forms structured storage units containing action name fields. This design facilitates subsequent behavior composition and execution, ensuring the flexibility of motion resources.

- A behavior script construction module based on temporal skill-chaining

This module transforms discrete action units into executable task sequences. The system constructs skill inheritance chains upon the completion of each action unit, enabling sequential playback and seamless transitions between actions. At the task sequence generation level, users combine action units via drag-and-drop operations within a visual editor, and the system automatically generates the corresponding behavior scripts.

- A behavior visualization module based on spatiotemporal synchronous mapping

Leveraging the 3D engine Blender, this module achieved synchronous co-evolution of the motion trajectories generated by the behavior planner along both temporal and spatial dimensions. Along the temporal dimension, actions were played sequentially according to their order on the timeline, clearly demonstrating the dynamic execution process of the behavior. Along the spatial dimension, the corresponding 3D motion postures were accurately reconstructed based on the specific parameters of each action unit.

**Figure 4:**
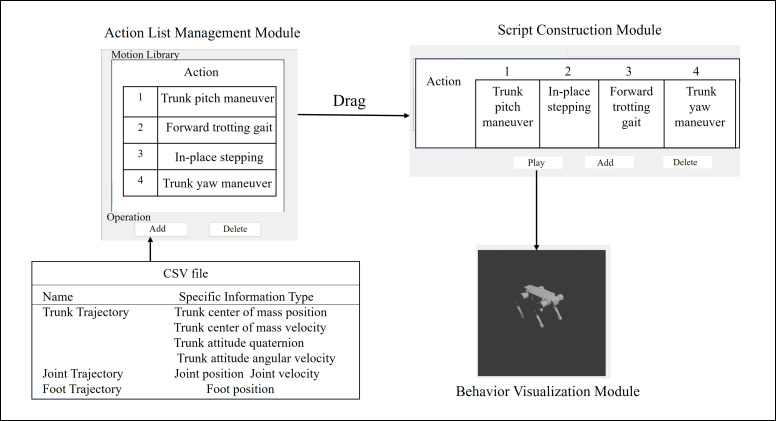
Overall workflow of the behavior planner.

The synergistic integration of the aforementioned modules formed a complete workflow, spanning from motion data import to behavior visualization. As illustrated in Figure 4, CSV files containing motion sequences were imported into the Action List Management Module via the “Add” option. Multiple motion sequences were then dragged into the Script Construction Module to form a more complex behavior sequence. Clicking “Play” subsequently generated the simulation animation within the Behavior Visualization Module.

### 2.2. Motion Control

This study formulates the motion imitation problem as a RL optimization task within the Markov Decision Process (MDP) framework, employing the Proximal Policy Optimization (PPO) algorithm. The core objective was to learn a control policy that enables the agent to maximize the expected return in a given task $J\left(\pi \right)$:

(3) $$J(\pi )=E_{\tau \sim p(\tau |\pi )}\left[\sum_{t=0}^{T-1}\gamma^{t}r_{t}\right]$$

where T denotes the time span of each episode, $\gamma \in \left[0,1\right]$ represents the discount factor, and $r_{t}$ indicates the instantaneous reward value. $p\left(\tau \left|\pi \right.\right)$ is the probability of trajectory τ under policy π.

#### 2.2.1. State and Action Space Design

To establish the imitation learning system architecture and to enable the efficient learning and generalization of complex motions by the policy network, the mathematical representation of the state space and action space must be addressed.

Within the quadruped robot imitation learning control system, the state space is structured based on the RL framework, integrating body IMU attitude features and joint motor feedback states. The normalized state vector is defined as $s_{t}=\left(q_{t-2:t},a_{t-3:t-1}\right)$, where $q_{t-2:t}$ represents the attitude angles over the previous three timesteps, primarily including the body roll, pitch, and yaw angles measured in real-time by the IMU and $a_{t-3\;:\;t-1}$ denotes the angular parameters of the 12 joint motors over the preceding three cycles. The action space adopts a joint position control strategy, with the target angles of the quadruped robot’s 12 degree-of-freedom (DOF) joints serving as the core representation. The action space is defined as a 12-dimensional continuous vector $a=\left[\theta_{1},\theta_{2},\ldots ,\theta_{12}\right]$, where each dimension corresponds to the target angle of a single joint.

#### 2.2.2. Design of Reward Functions Based on Multi-Task Learning

This paper constructs a hierarchical and progressive reward function framework based on the statistical features of expert demonstration data and the kinematic–dynamic characteristics of robots. It enforces the policy to replicate expert actions through trajectory tracking reward terms; introduces motion stability reward terms to mitigate the risk of instability under disturbances; and guides the policy to achieve safety assurances on the foundation of imitation via safety reward terms. The mathematical expression for the reward at each time step in the reward function was defined as follows:

(4) $$r_{t}=r_{t}^{track}+r_{t}^{stab}+r_{t}^{safe}$$

where $r_{t}^{track}$ denotes the trajectory tracking reward function, $r_{t}^{stab}$ represents the motion stability reward function, and $r_{t}^{safe}$ is the safety reward function.

The trajectory tracking reward function quantifies the similarity between policy-generated motion trajectories and expert demonstration data through mathematical measurement, thereby providing explicit gradient signals for policy optimization. Its specific mathematical expression is defined as follows:

(5) $$r_{t}^{track}=\omega^{p}r_{t}^{p}+\omega^{r}r_{t}^{r}+\omega^{vl}r_{t}^{vl}+\omega^{va}r_{t}^{va}+\omega^{e}r_{t}^{e}+\omega^{q}r_{t}^{q}+\omega^{qv}r_{t}^{qv}$$

The constituent reward terms are defined as follows: centroid position reward $r_{t}^{p}$, centroid orientation reward $r_{t}^{r}$, centroid linear velocity reward $r_{t}^{vl}$, centroid angular velocity reward $r_{t}^{va}$, foot-end position reward $r_{t}^{e}$, joint position reward $r_{t}^{q}$, and joint velocity reward $r_{t}^{qv}$. Each reward function adopts the exponential quadratic form $r=\exp\left[-\alpha {\left\|\hat{x}-x\right\|}^{2}\right]$, where tunable hyperparameter α regulates the reward sensitivity. Here, *x* and $\hat{x}$ denote the actual state and desired expert state, respectively.

The motion stability reward function mathematically quantifies deviations between the robot’s torso attitude, centroid motion state, and stable equilibrium objectives, thus guiding the policy network to generate disturbance-resistant motion patterns. Its comprehensive expression is defined as follows:

(6) $$r_{t}^{stab}=\lambda_{\mathrm{ori}}\cdot \cos\left({\hat{z}}_{B},{\hat{z}}_{W}\right)-\lambda_{\mathrm{com}}\cdot d_{\mathrm{com}}$$

where ${\hat{z}}_{B},{\hat{z}}_{W}$ represents the torso attitude angles and the gravity-aligned *z*-axis unit vector, $d_{\mathrm{com}}$ indicates the projection distance of the centroid within the support polygon, and $\lambda_{\mathrm{ori}},\lambda_{\mathrm{com}}$ are the corresponding weighting coefficients.

The safety reward function enforces mathematical constraints to ensure critical parameters, including the joint positions, velocities, and torques of output actions, which consistently remained within physical viability and system safety thresholds.

(7) $$r_{t}^{safe}=-\left(\sum_{i=1}^{12}\left[k_{p}\cdot {\left(\theta_{i}-\theta_{\mathrm{threshold}}\right)}^{2}+\exp\left(\frac{\left|{\dot{\theta }}_{i}\right|}{{\dot{\theta }}_{\max}}\right)-1+\lambda_{\tau }\cdot {\left(\frac{\left|\tau_{i}\right|}{\tau_{\max}}\right)}^{3}\right]\right)$$

where $\theta_{threshold}=\theta_{\min}+\delta$ denotes soft constraint thresholds, δ=0.1rad represents a buffer zone to prevent policy oscillation induced by abrupt clamping, ${\dot{\theta }}_{\max}$ and $\tau_{\max}$ specify the maximum allowable joint velocity and torque, respectively, and $k_{p}$, $\lambda_{\tau }$ are corresponding proportional coefficients.

#### 2.2.3. Imitation Learning Network

Integrating all components of the imitation learning control system yielded the overall architecture of the quadruped robot imitation learning controller. This framework is primarily divided into four components: Actor network, Critic network, low-level controller, and policy optimization.

**Figure 5:**
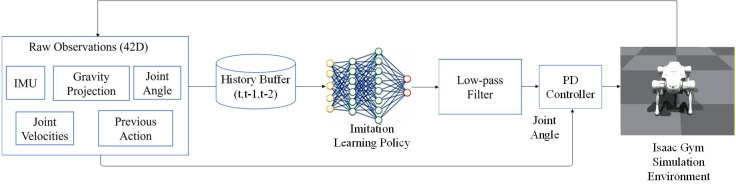
Imitation Learning Policy Controller.

The Actor network module enables the controller to regulate the physical model of robots. As depicted in Figure 5, the Actor network accepts 42-dimensional input vectors consisting primarily of raw environmental observations, specifically selected as $\left[v_{ang},G,q_{j},{\dot{q}}_{j},a_{last}\right]\in R^{42}$. The information is typically input into the policy network in the form of low-dimensional vectors, which accelerate the training process and improve the convergence efficiency of the policy. The complete set of observations is shown in Table 1. Within the Isaac Gym training environment, body pose sensors, historical joint sensors, and joint position sensors were implemented to collect raw observational data. These observations were subsequently stored in a history buffer, where data from each sensor were packaged across three consecutive timesteps (t, t − 1, t − 2) and sequentially concatenated to serve as inputs for the imitation learning policy.

**Figure 6:**
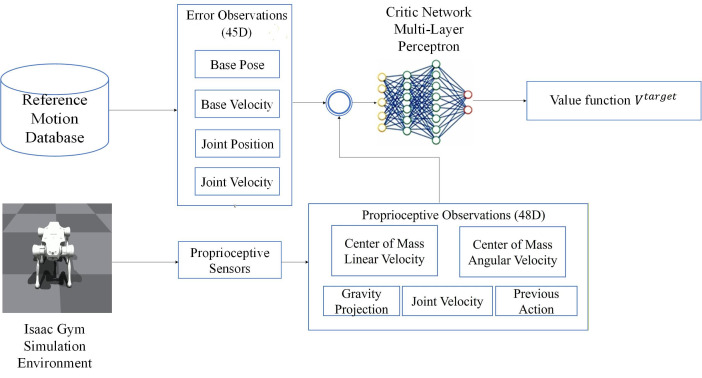
Control Framework of Critic Network.

The Critic network module must balance efficient representation of dynamic environments with stability requirements for policy optimization, as illustrated in the control framework (Figure 6). For quadruped robot locomotion control, the Critic network’s input feature space comprises $\left[v_{lin},v_{ang},G,q_{j},{\dot{q}}_{j},a_{last},e_{errot}\right]\in R^{93}$. The details of the tracking error are presented in Table 2, typically including proprioceptive information and task-specific high-level goal parameters. Unlike the Actor network that relies solely on current state observations, the Critic network explicitly models the dynamic characteristics of goal deviation during policy execution by incorporating tracking error information, thereby introducing task-oriented long-term reward mechanisms into value estimation.

The low-level controller converts joint position increments from the Actor network into desired rotation commands. These commands are processed by a Proportional-Derivative (PD) controller to compute joint torques for motor actuation, with the control torque formulated as follows:

(8) $$u(t)=K_{p}\cdot e(t)+K_{d}\cdot \frac{de(t)}{dt}$$

where $K_{p}$ and $K_{d}$ represent the proportional and derivative gains, respectively, e(t) denotes position error, and de(t)/dt indicates velocity error. To simplify control objectives and enhance stability, the desired joint velocity was typically set to zero.

PPO is an optimization algorithm based on the Actor–Critic architecture, whose core lies in the update mechanisms for the policy and value networks. Upon receiving control commands, the agent utilizes current state information to compute the instantaneous reward signal R through the reward function. The Critic network outputs an estimate of the state-value function $V^{target}$. This estimate is combined with R to compute the advantage estimate and quantify the discrepancy between the value function prediction and the actual reward, thereby providing comprehensive feedback for policy evaluation.

The advantage estimation adopts the form of generalized advantage estimation (GAE), denoted as ${\hat{A}}_{t}^{\mathrm{GAE}}$. This method employs an adjustable parameter λ to perform an exponentially weighted fusion of Temporal Difference (TD) errors across different step lengths, establishing a continuously tunable balance mechanism between estimation bias and variance. The K-step TD error $\delta_{t}^{V}$ is defined as follows:

(9) $$\delta_{t}^{V}=r_{t}+\gamma V(s_{t+1})-V(s_{t})$$

where $v\left(s\right)$ represents the state-value function, γ denotes the discount factor, and t is the timestep index. GAE computes an exponentially weighted average of K-step TD errors, so a compact form of GAE ${\hat{A}}_{t}^{\mathrm{GAE}}$ can be derived through series expansion, as follows:

(10) $${\hat{A}}_{t}^{\mathrm{GAE}}=\sum_{l=0}^{T-t}{\left(\gamma \lambda \right)}^{l}\delta_{t+l}^{V}$$

The statistical properties of GAE are jointly determined by the discount factor γ and λ. γ controls the discounting of future returns, influencing the focus on long-term gains. λ regulates the bias–variance trade-off intensity. In most continuous control tasks, setting λ = 0.95 yields empirically optimal performance.

The Critic network takes proprioceptive observations and error observations as inputs. After processing through a three-layer neural network, it outputs the estimated state function $V^{target}$ for the given state. The objective is to approximate $V^{target}$, providing a benchmark for policy evaluation and subsequently guiding the policy updates of the Actor network. The update of the Critic network relies on the accurate estimation of $V^{target}$, aiming to minimize the mean squared error between the predicted values and the target values. The target value is computed using the TD (λ) method, which extends Temporal Difference (TD) learning by introducing eligibility traces to assign credit for rewards. The TD (λ) update rule is given by the following:

(11) $$V\left(s_{t}\right)\leftarrow V\left(s_{t}\right)+\alpha \delta_{t}E_{t}$$

where $V\left(s_{t}\right)$ is the estimated value of the current state, α is the learning rate, $\delta_{t}$ is the TD error at timestep *t*, and $E_{t}$ is the eligibility trace at timestep t. This process drives the value function toward the true value by minimizing the mean squared error between the predicted and target values.

#### 2.2.4. Domain Randomization and Disturbances

To improve robustness against sim-to-real discrepancies and cross-source motion variations, we incorporated domain randomization and observation noise injection during training.

Specifically, key physical parameters, including mass, inertia, actuator-related parameters, and friction coefficients, were randomly perturbed within predefined ranges, as summarized in Table 3. In addition, random offsets were applied to the initial states, including joint configurations and Center of Mass (CoM) states, to account for initialization uncertainties.

Furthermore, observation noise was injected to model measurement noise and observation uncertainty. The noise magnitudes applied to joint states, CoM velocities, and gravity projection are listed in Table 4. These randomization and noise injection strategies collectively improved the stability and generalization of the learned whole-body control policy under uncertainties.

## 3. Experimental Tests and Results

This study employed the Lite3 quadruped robot model developed by DeepRobotics (Hangzhou, China), conducting training on the Isaac Gym simulation platform with an NVIDIA GeForce RTX 4090 GPU. Subsequently, the trained policy underwent dynamic adaptability validation for the robot model in the PyBullet physics engine. Additionally, the Blender 4.1 3D animation engine was utilized to create quadruped robot motion animations via keyframe insertion, facilitating motion sequence design and export. For RL, the PPO algorithm was adopted due to its computational efficiency. The hyperparameter configurations used during PPO-based simulation training are summarized in Table 5.

In the experiments, the centroid height of the quadruped robot in standing posture was set to 0.32 m. The joint angle vector was initialized to $q={\left[0,\;-0.773,\;1.5\right]}^{T}$ for hip, thigh, and calf joints, respectively. To ensure that joint rotation remained within safe operating limits, the upper and lower bounds of the joint motion range are detailed in Table 6.

### 3.1. Motion Design Validation Using a 3D Engine

**Figure 7:**
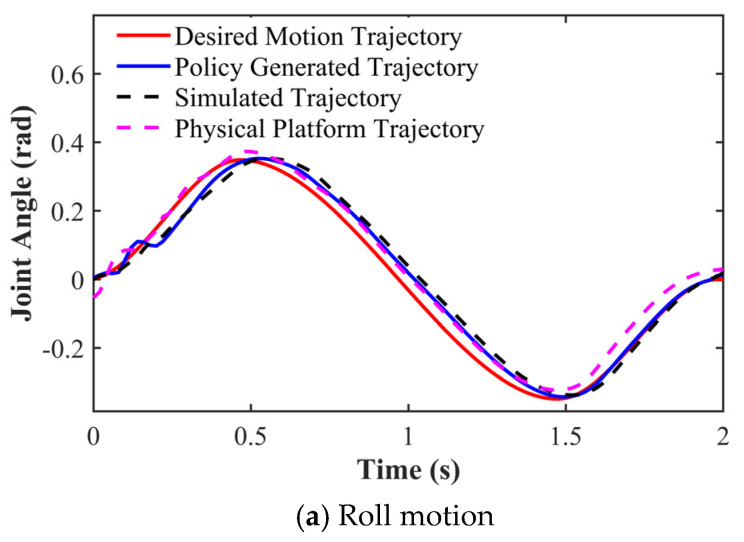

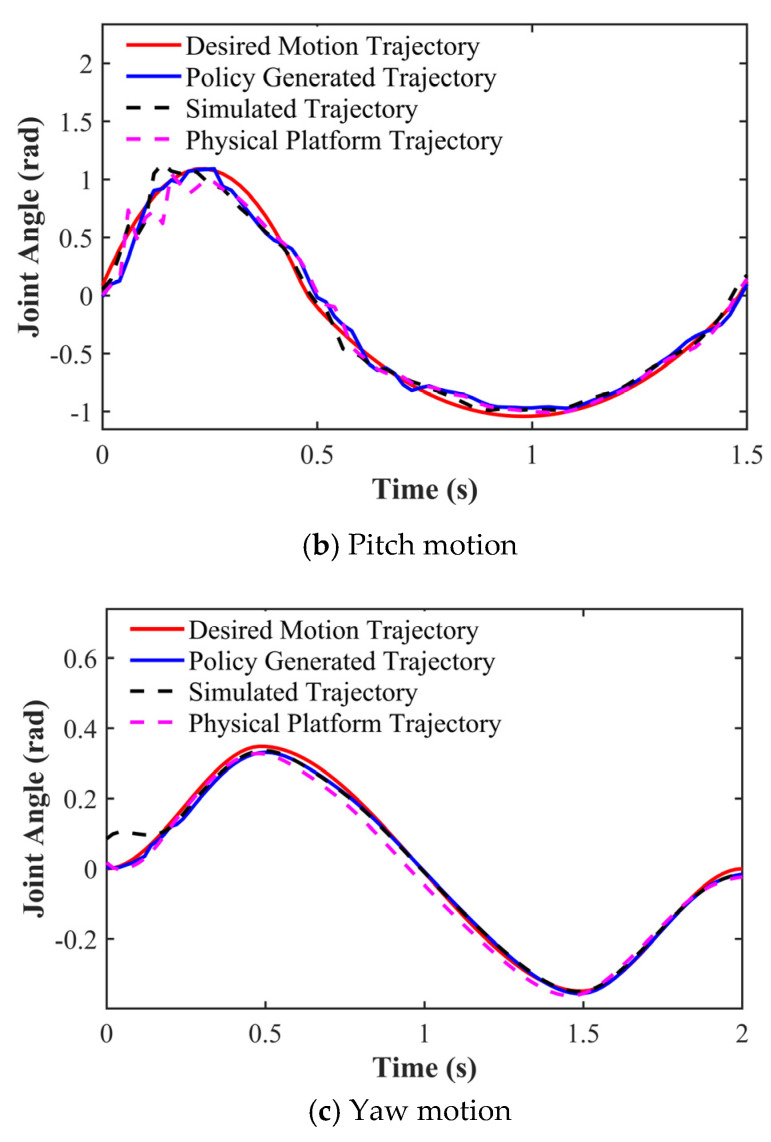
Euler angle tracking performance of the robot’s Center of Mass (COM). The curves compare the desired trajectories with the policy-generated, simulated, and physical platform responses for (**a**) Roll, (**b**) Pitch, and (**c**) Yaw motions.

The experiment utilized Blender to create motion sequences, incorporating roll, pitch, and yaw rotations, with target angles set at ±20° in both directions. Motion control policies were generated in parametric space through imitation learning, with trajectory tracking accuracy verified via PyBullet simulation and the Lite3 physical platform. Figure 7 presents comparative results of Euler angle tracking performance. Quantitative analysis shows the maximum tracking errors of 0.03 rad (roll), 0.01 rad (pitch), and 0.01 rad (yaw), meeting precision requirements for motion control.

### 3.2. Experimental Validation of Motion Capture-Based Motion Repositioning

**Figure 8:**
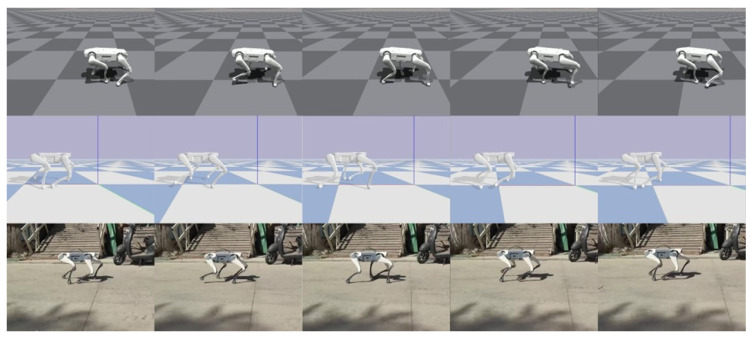
Repositioned trot gait snapshots. From top to bottom, the snapshots correspond to Isaac Gym, PyBullet, and real-world environments, respectively.

**Figure 9:**
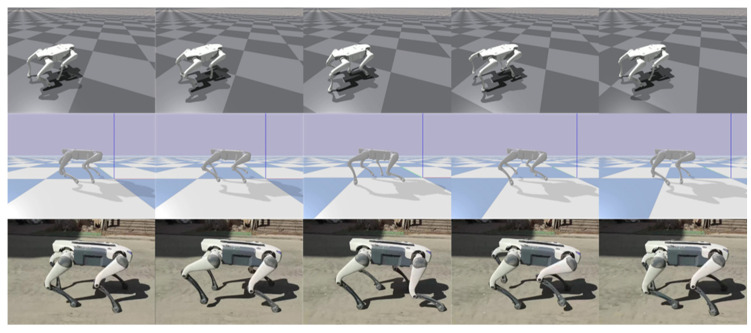
Snapshots of repositioned backward trot motion. From top to bottom, the snapshots correspond to Isaac Gym, PyBullet, and real-world environments, respectively.

In quadruped motion repositioning research, we first acquired the reference trajectory data adapted for the Lite3 robot by performing inverse kinematic mapping on motion-captured trot gait patterns, validating the effectiveness of forward trot motion (whose action sequence is shown in Figure 8). Subsequently, through inverse kinematics parameter inversion, a mirrored backward trot motion sequence and control strategy were generated (with motion snapshots presented in Figure 9).

**Figure 10:**
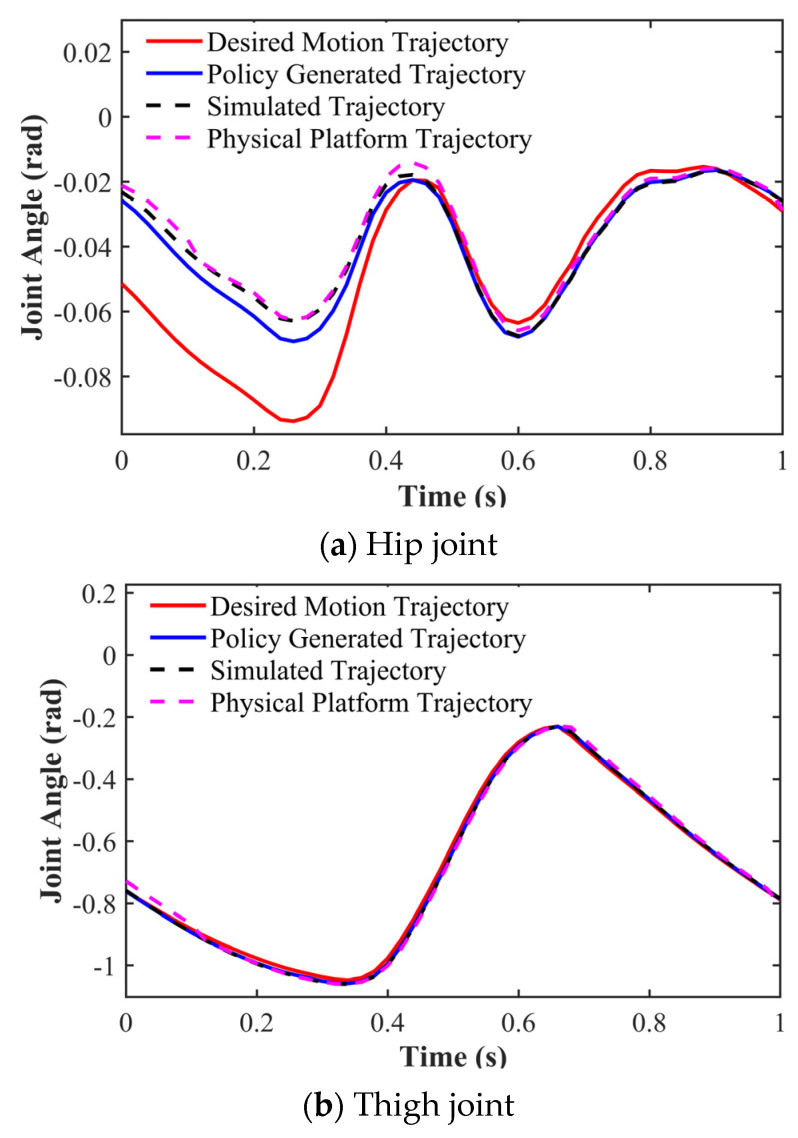

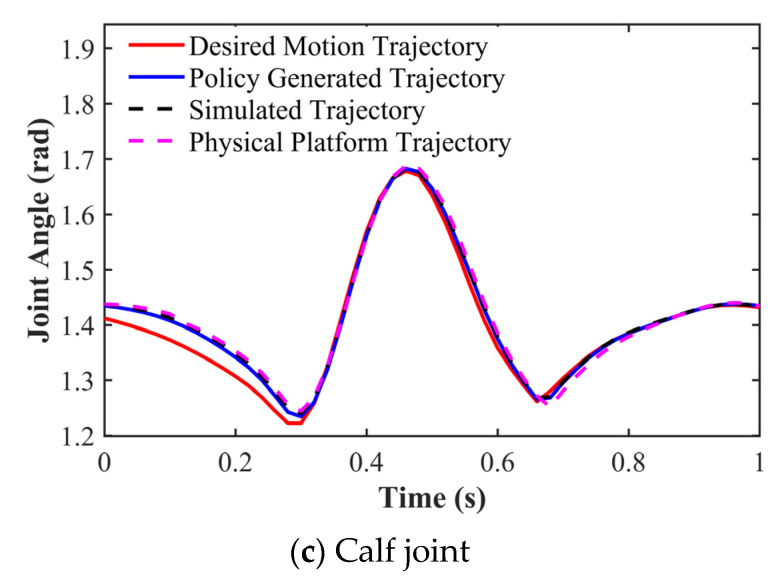
Joint angle tracking curves of the right foreleg.

**Figure 11:**
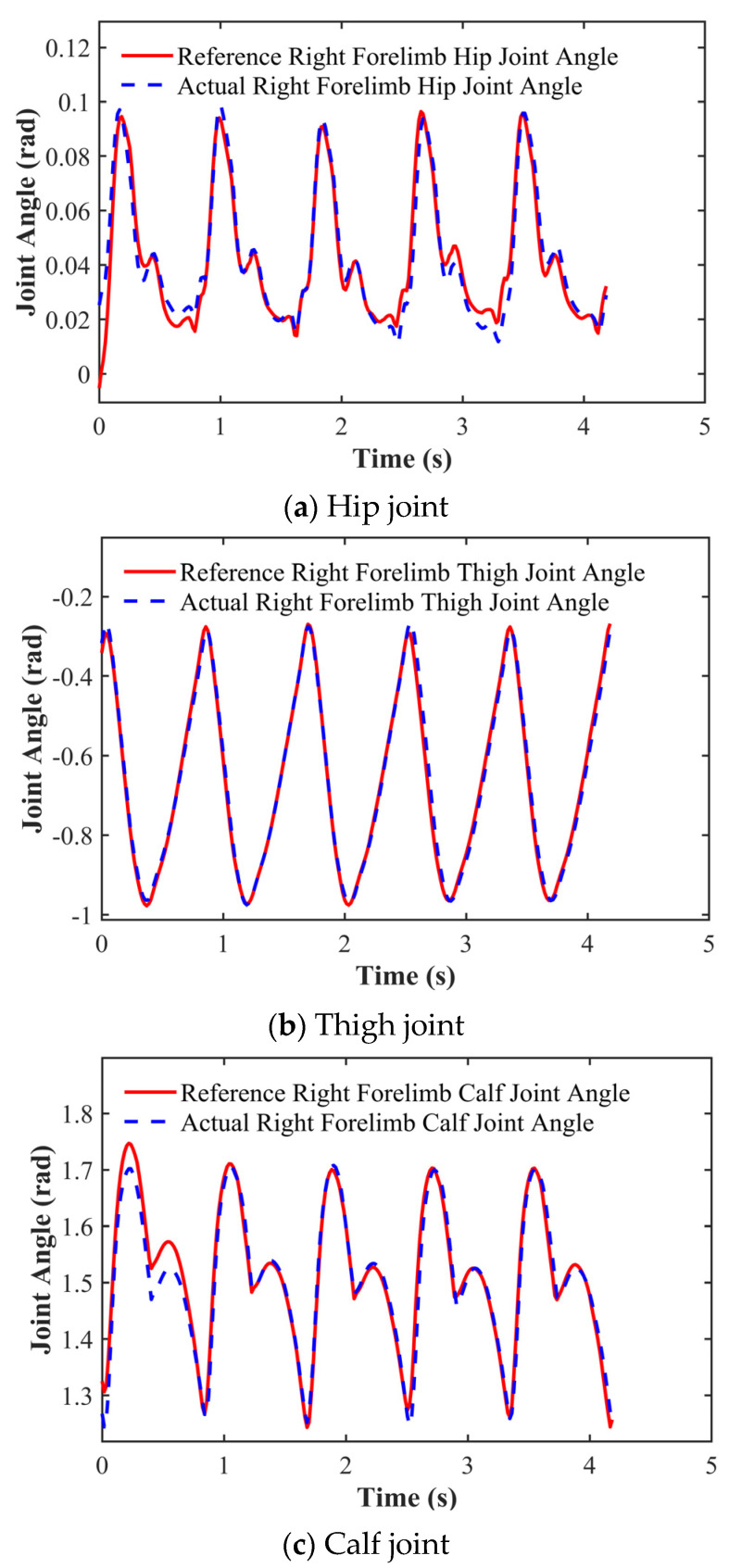
Joint angle variation curves of the right foreleg.

We recorded the three DOF joint rotation angles in the robot’s right forelimb. As illustrated in Figure 10, the complete gait cycle analysis demonstrates that trained, simulated, and experimentally measured trajectories all effectively track target joint angles, while exhibiting excellent continuity and smoothness without observable step distortion. Notably, the hip joint’s initial rotation displays an inward flexion tendency due to training-phase configurations designed to ensure motion initiation continuity. Figure 11 further presents the joint angle tracking performance of the hip, thigh, and calf joints during 5 s periodic motions, with quantitative data confirming the effective reference angle across all three joints.

### 3.3. Sim-to-Real Deployment and Experimental Verification

To evaluate sim-to-real transfer performance, the policy trained in simulation was directly deployed on the Lite3 quadruped robot without any additional fine-tuning. During training, limited domain randomization and observation noise were introduced to improve robustness to real-world uncertainties, as described in Section 2.2.4.

After training converges, the PyTorch 1.13.1 actor network was serialized into a static TorchScript model (.pt) and transferred from the training workstation to the robot’s onboard computer (NVIDIA Jetson Orin NX) via a secure network interface (SSH). Onboard, the policy was loaded using C++/LibTorch for online inference, with observation normalization and state processing kept consistent with simulation.

The real-world control system follows a low-rate policy inference–high-rate execution architecture. The policy runs at 50 Hz and outputs joint position residuals, which are mapped to target joint positions and tracked by a 1 kHz low-level PD controller. A state-based safety failsafe was implemented to ensure hardware safety during experiments.

To quantitatively assess sim-to-real transfer fidelity, simulated (Sim) and real-world (Real) trajectories were compared under identical command inputs. The discrepancy between simulation and real execution was evaluated using three standard metrics: root mean square error (RMSE), mean absolute error (MAE), and maximum absolute error (Max Error).

Specifically, errors of the robot CoM attitude, represented by Roll, Pitch, and Yaw, are summarized in Table 7. In addition, joint-level errors for Hip, Thigh, and Knee are reported in Table 8 to characterize sim-to-real discrepancies at the actuator execution level.

The quantitative errors reported in Table 7 and Table 8 are computed from the corresponding tracking trajectories presented in the subsequent experimental results. In particular, the CoM attitude errors are derived from the yaw motion shown in Figure 7, whereas the joint-state errors are calculated based on the right foreleg joint trajectories shown in Figure 10 and Figure 11.

As shown in Table 7 and Table 8, the discrepancies between simulated and real robot executions remain within a small and bounded range across all reported metrics. Specifically, the RMSE and MAE of the robot CoM Euler angles were below 0.1 rad, whereas the corresponding maximum absolute errors remain limited. Similarly, for key joint states, both RMSE and MAE remain below 0.04 rad, with bounded maximum errors. Overall, these results indicate that the simulation results closely reflect the execution behavior on real hardware.

### 3.4. Experimental Validation of CPG-Based Trajectory Planning Motion Design

**Figure 12:**
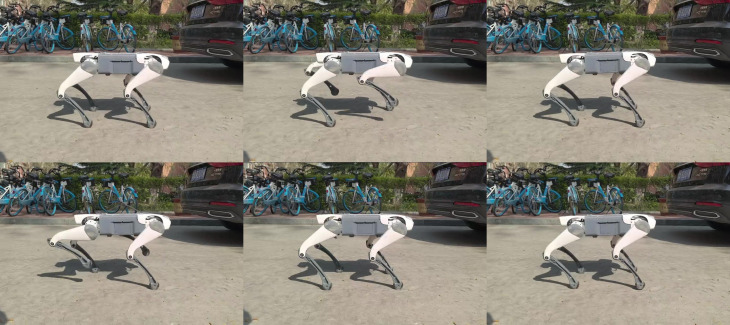
Quadruped robot in-place stepping experiment.

Taking the CPG-generated in-place stepping motion as an example, a phase-coupled oscillator model was employed to establish parametric equations for foot-end trajectories, with a gait cycle of 0.5 s and a leg lift height of 0.1 m. Figure 12 demonstrates the physical control performance, where the robot accurately replicates reference trajectories, validating the framework’s effectiveness for CPG-planned motions. This controller maintains consistency with the previously described Blender and motion capture-based controllers.

### 3.5. Multi-Action Composite Behavior Experiment

**Figure 13:**
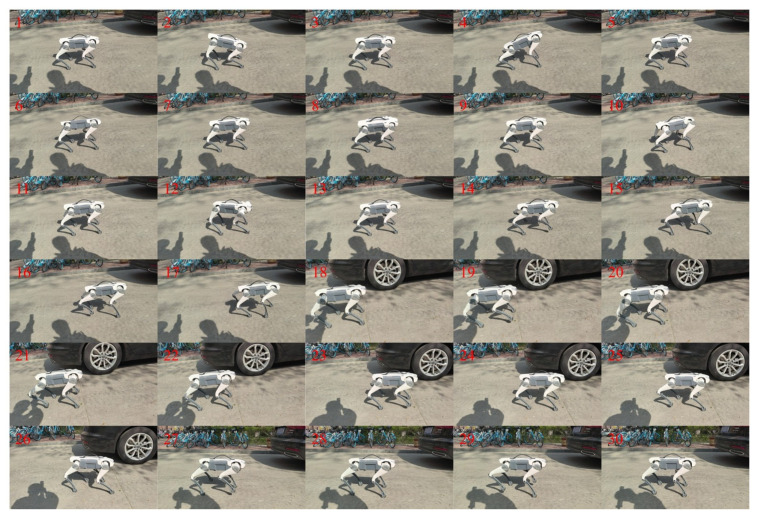
Multi-task control implementation snapshots.

Building upon the validated single-task control architecture, we developed an imitation learning-based multi-task control system. This system integrates Blender-generated torso twisting, motion-captured trot repositioning (forward/backward), and CPG-based in-place stepping into a unified behavioral dataset. Figure 13 presents the physical robot executing this multi-action sequence, visually confirming the imitation learning framework’s capability for composite behavior generation.

### 3.6. Ablation Study on Temporal Behavior Planning

To isolate the contribution of the proposed temporal behavior planner, we conducted an ablation study, in which all compared methods shared the same imitation-learning-based low-level controller. All methods were evaluated under identical dataset splits, experimental settings, evaluation metrics, and on the same physical hardware platform, ensuring a fair and controlled comparison. We compared our behavior-planner-based motion primitive transition approach with two motion switching strategies commonly used in practice:

Method A: direct switching, where motion primitives are concatenated without any temporal smoothing or state alignment;

Method B: reset-to-neutral execution, where the robot returns to a stable standing pose and waits for stabilization before executing the next primitive [18,28];

Method C: continuous behavior planning (ours), which explicitly synthesizes smooth transition trajectories in the temporal domain.

**Figure 14:**
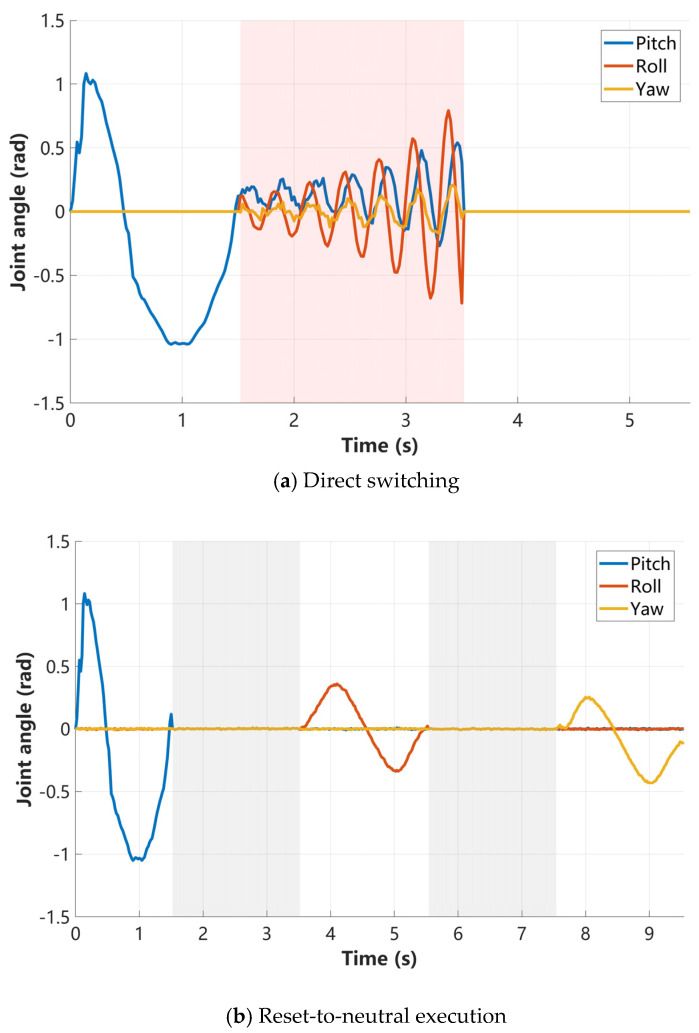

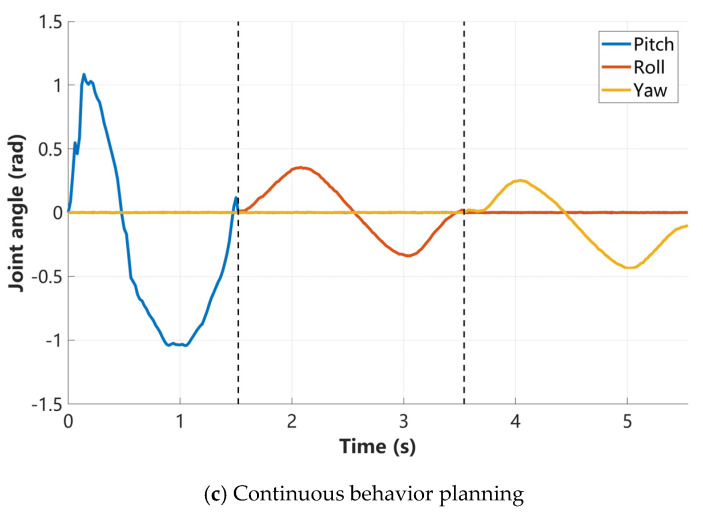
Attitude response comparisons under different motion primitive transition strategies.

Figure 14 shows the variation of the robot’s CoM Euler angles under different motion switching strategies. As shown in Figure 14a, Method A lacks effective continuity when transitioning from the previous motion to the next, resulting in poorly controlled attitude changes and preventing the robot from successfully completing subsequent actions. As shown in Figure 14b, Method B restores the robot to an initial standing posture before executing the next motion, enabling the completion of the entire motion sequence.

In contrast, as shown in Figure 14c, Method C (ours) maintains continuity between consecutive motions during switching, keeping the attitude changes smooth. The roll, pitch, and yaw angles vary smoothly over time, allowing the motion sequence to be completed continuously.

Overall, although both the proposed method and the reset-based baseline were able to complete the task without instability, our approach achieves substantially smoother execution and higher efficiency by eliminating unnecessary waiting phases. In contrast, direct switching consistently fails due to the lack of effective continuity between consecutive motions.

Since all methods employ the same low-level controller, the observed performance differences can be solely attributed to the motion primitive transition strategy. These results suggest that the temporal behavior planner is beneficial for achieving stable, continuous, and efficient multi-skill execution on real robotic systems.

## 4. Conclusions

This paper proposes a quadruped robot motion strategy generation method based on multimodal motion primitives and imitation learning. The method constructs a three-level architecture of “motion primitives–skill chains–complex behaviors”, enabling parameterized design under joint constraints, biological feature transfer, and tunable trajectory generation. Meanwhile, a multi-task control framework based on imitation learning was designed, which adopts the Actor–Critic architecture and a hierarchical composite reward function. A training–simulation–deployment verification system was built based on Blender and physical entity platforms, systematically verifying the generation of basic gaits and the effect of motion transfer, as well as the execution efficiency and generalization ability of dynamic behavior sequences under multi-task control.

It should be emphasized that the proposed method was evaluated under structured task settings with predefined motion primitives and controlled execution conditions. Although the results confirm its capability in generating and composing multimodal motions, its performance in highly dynamic tasks and unstructured environments has not yet been systematically investigated. Future work will focus on incorporating dynamics-aware optimization and robustness enhancement to extend the applicability of the framework to more complex real-world scenarios.

## Figures and Tables

**Table 1:** Observations of the Policy Network.

Symbol	Dimension	Description
$v_{ang}$	3	Angular velocity of the center of mass (COM)
G	3	Projection of gravitational force
$q_{j}$	12	Joint angle
${\dot{q}}_{j}$	12	Joint velocity
$a_{last}$	12	Action at the previous time step

**Table 2:** Tracking Errors in the Privileged Observations.

Symbol	Dimension	Description
CoM_pos_error	3	Tracking error of COM’S position, relative to the world coordinate system
CoM_euler_error	3	Tracking error of COM’s Euler angle
CoM_lin_error	3	Tracking error of COM’s linear velocity
CoM_ang_error	3	Tracking error of COM’s angular velocity
toe_pos_error	12	Tracking error of COM’S position, relative to the world coordinate system
dof_pos_error	12	Joint position tracking error
dof_vel_error	12	Joint velocity tracking error

**Table 3:** Domain randomization of physical parameters.

Item	Range
Mass	[0.8, 1.2] × default
Inertia	[0.5, 1.5] × default
Motor torque scaling	[0.8, 1.2] × default
PD gain scaling	[0.8, 1.2] × default
Initial joint position scaling	[0.5, 1.5] × default
Initial CoM position scaling	[0.8, 1.2] × default
Ground friction	[0.1, 2.5]
Motor friction	[0, 0.05]

**Table 4:** Observation noise settings.

Item	Noise Magnitude
Joint position noise	0.01
Joint velocity noise	1.5
CoM linear velocity noise	0.1
CoM angular velocity noise	0.2
Gravity projection noise	0.05

**Table 5:** Hyperparameter configurations for PPO during simulated training.

Parameter	Value
Learning rate	${2\times 10}^{-5}/{1\times 10}^{-5}$
Discount factor γ	0.99
Batch size	98,304(4096×24)
Mini-batch size	24,576(4096×6)
Policy clip range	0.2
Entropy coefficient	0.01
GAE λ	0.95
Target KL divergence	0.01

**Table 6:** Joint motion range contraints.

Joint Type	Dimension	Thigh Joint (rad)	Calf Joint (rad)
Upper limit	0.523	0.314	2.792
Lower limit	−0.523	−2.67	0.524

**Table 7:** Quantitative Sim-to-Real Errors of Robot CoM Euler Angles.

Attitude	RMSE (rad)	MAE (rad)	Max Error (rad)
Roll	0.0398	0.0343	0.0763
Pitch	0.0974	0.0575	0.5145
Yaw	0.0315	0.0226	0.1075

**Table 8:** Quantitative Sim-to-Real Errors of Robot Joint States.

Joint	RMSE (rad)	MAE (rad)	Max Error (rad)
Hip	0.0057	0.0043	0.0146
Thigh	0.0386	0.0331	0.0830
Knee	0.0324	0.0230	0.0872

## Data Availability

The original contributions presented in this study are included in the article material. Further inquiries can be directed to the corresponding author.
